# Acute abdominopelvic pain and distension in a 21-year-old woman revealing a mixed germ cell tumor: A case report

**DOI:** 10.1016/j.ijscr.2025.111063

**Published:** 2025-02-16

**Authors:** Dhekra Toumi, Chayma Cheikh Mohamed, Imen Ghaddab, Amina Ben Salem, Ahlem Bellalah, Ahmed Hajji

**Affiliations:** a Faculty of Medicine of Monastir, Department of Gynecology and Obstetrics, Fattouma Bourguiba University Hospital, Monastir, Tunisia; b Faculty of Medicine of Monastir, Department of Radiology, Fattouma Bourguiba University Hospital, Monastir, Tunisia; c Faculty of Medicine of Monastir, Department of Anatomical Pathology, Fattouma Bourguiba University Hospital, Monastir, Tunisia

**Keywords:** Primary ovarian germ cell tumors, Young woman, Unilateral adnexectomy, Cryo preservation, Chemotherapy sensitivity

## Abstract

**Introduction and importance:**

Primary ovarian germ cell tumors (OGCTs) account for 5 % of malignant pelvic neoplasms, predominantly affecting young females and often presenting as medical emergencies. Among OGCTs, malignant mixed germ cell tumors constitute approximately 8 % of cases and are characterized by their aggressive nature. Early and appropriate management of these rare tumors is crucial for optimizing patient outcomes.

**Case presentation:**

We present the case of a 21-year-old female who was admitted urgently due to a large abdominopelvic mass. Clinical evaluation and supplementary investigations confirmed the diagnosis of a mixed OGCT, featuring the most common combination of yolk sac tumor and dysgerminoma. A transvaginal surgical approach was employed to preserve the patient's hymenal integrity and facilitate oocyte preservation, in accordance with her cultural and personal beliefs. The treatment consisted of unilateral adnexectomy followed by platinum-based chemotherapy.

**Clinical discussion:**

Mixed OGCTs are rare but highly aggressive tumors that primarily affect young women. Despite their aggressive nature, these tumors often have a favorable prognosis when promptly diagnosed and treated. This case highlights the importance of individualized, fertility-preserving approaches in young patients. The combination of yolk sac tumor and dysgerminoma necessitated a multimodal treatment strategy to achieve optimal outcomes while respecting the patient's reproductive aspirations.

**Conclusion:**

Mixed OGCTs are uncommon neoplasms that require early diagnosis and tailored management. This case is noteworthy due to the rare presentation of a mixed germ cell tumor in a young woman and the multidisciplinary approach undertaken to balance oncological management with fertility preservation, utilizing a transvaginal surgical technique to respect the patient's cultural and personal beliefs.

## Highlights

• The extremely rare occurrence of mixed germinal ovarian tumors
• Adnexectomy with adjuvant platinum-based chemotherapy is the gold standard of treatment.
• Compliance with patient conventions for fertility preservation
• Efficacy of adnexectomy with adjuvant platinum-based chemotherapy
• The contribution of serum markers to follow-up

## 1 Introduction

Primary ovarian germ cell tumors (OGCTs) account for 5 % of malignant pelvic neoplasms; they are rare, highly aggressive, yet treatable tumors [1]. These neoplasms predominantly affect young females and are characterized by rapid growth, often leading to their detection in emergency settings or as acutely complicated cases. Initial treatment is typically surgical, with conservative methods prioritized whenever feasible to preserve future fertility.

These tumors are highly chemosensitive and produce specific tumor markers that aid in both diagnosis and therapeutic monitoring [1]. OGCTs exhibit histological diversity, originating from embryonic gonadal germ cells [2]. The World Health Organization classifies malignant germ cell tumors into dysgerminoma, endodermal sinus (yolk sac) tumor, immature teratoma, non-gestational choriocarcinoma, embryonal carcinoma, and mixed germ cell tumors [3]. By definition, malignant mixed germ cell tumors contain two or more malignant germ cell types. These tumors are exceedingly rare, accounting for 8 % of OGCT cases, and are notably aggressive [4].

Here, we present a case of a malignant mixed germ cell tumor of the ovary, involving the common combination of yolk sac tumor and dysgerminoma. This report details its clinical, biological, radiological, therapeutic, and prognostic aspects. This case report has been prepared in accordance with the SCARE guidelines [5].

## 2 Patient presentation

A 21-year-old Caucasian female presented to our emergency department with progressive abdominopelvic pain and abdominal distension. Her symptoms had gradually worsened over the past seven months, during which she had noticed a steady increase in abdominal volume but had not sought medical attention.

On physical examination, the patient was hemodynamically stable. Abdominal palpation revealed a large, firm, and immobile abdominopelvic mass extending from the left iliac fossa to the lower hepatic border. No signs of peritoneal irritation or acute distress were observed.

### 2.1 Diagnostic approach

**Fig. 1:**
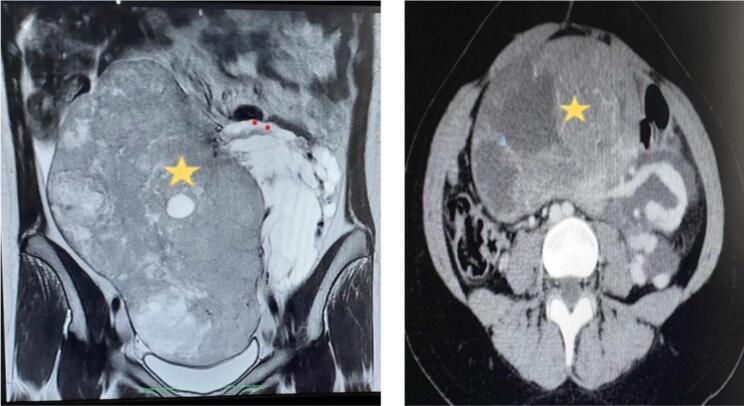
Abdominopelvic MRI. (star) A large, oblong, right paramedian, solid-cystic abdominopelvic mass with predominant solid areas showing mild hyperintensity on T2, hypointensity on T1, hyperintensity on diffusion with restricted diffusion, and enhancement after gadolinium injection.

Initial imaging with an abdominopelvic ultrasound revealed a heterogeneous tissue echogenicity with anechoic halos extending beyond the screen limits, prompting further evaluation with pelvic MRI. MRI findings demonstrated a large, oblong, right paramedian solid-cystic abdominopelvic mass with a solid component exhibiting slight T2 hypersignal, T1 hyposignal, and diffusion hypersignal with diffusion restriction, showing enhancement post‑gadolinium injection. The mass measured 171 × 100 mm in the axial view and 227 mm in the sagittal view, most likely originating from the left ovary and classified as ORADS 5. Additionally, mild to moderate intraperitoneal effusion was observed, along with a left-sided loculated effusion encasing the ipsilateral ovarian pedicle, raising suspicion of carcinomatosis (Fig. 1).

**Fig. 2:**
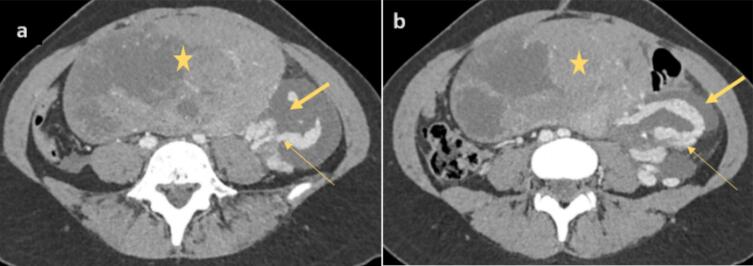
(a) and (b): CT slices at the abdominal level showing a mass (star) effect on the left ovarian pedicle with tortuous dilation of the ipsilateral ovarian vein (large arrow) and a lymphocele (small arrow).

A staging CT scan revealed no evidence of secondary metastases but confirmed that the mass was in close contact with the small intestine, displacing it anteriorly, and the sigmoid colon, displacing it posteriorly, without any signs of bowel wall thickening or digestive tract distension. On the mass's left lateral aspect, a significant mass effect was observed on the left ovarian pedicle, accompanied by tortuous dilation of the ipsilateral ovarian vein, which remained patent. Additionally, a multilobulated fluid collection surrounding the ovarian pedicle extended retroperitoneally up to the left renal pedicle and approached the left side of the aorta, suggesting the presence of a lymphocele with minimal intraperitoneal effusion (Fig. 2).

**Fig. 3:**
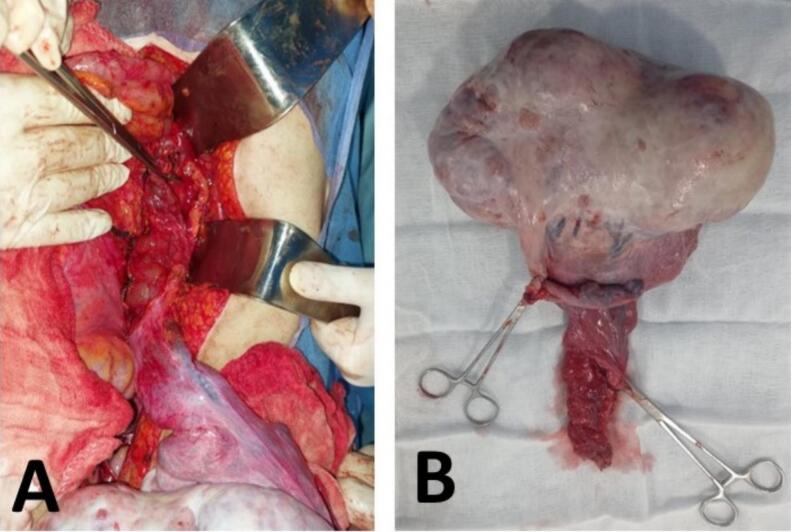
(A): Intraoperative lymphocele. (B): Specimen after left adnexectomy.

Tumor marker analysis revealed elevated alpha-fetoprotein (AFP) at 6371 IU/mL and beta-hCG at 71.2 IU/mL. Given these findings, the multidisciplinary team opted for an exploratory laparotomy. Intraoperatively, moderate ascites was observed, along with a 30 cm encapsulated mass originating from the left ovary. The mass exhibited a firm, mixed consistency with irregular contours and extensions reaching into the retroperitoneal space, encasing the left renal vein (Fig. 3).

Cytological analysis was performed. Surgical management involved a left adnexectomy with meticulous dissection of the lumborenal-ovarian extension. Critical structures in the left renal fossa were carefully preserved, and the integrity of the ureter and renal pelvis was confirmed. Additionally, staged peritoneal biopsies were obtained for further evaluation.

### 2.2 Follow-up and outcomes of therapeutic interventions

**Fig. 4:**
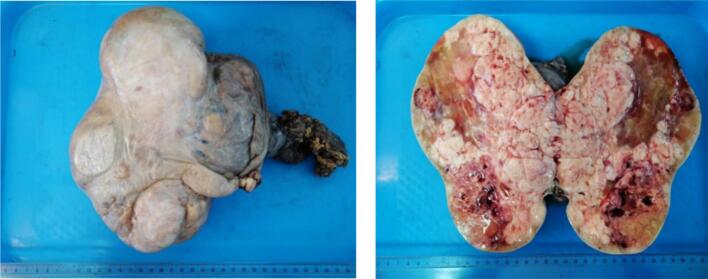
Macroscopic examination of the pathology specimen.

Final histopathological analysis revealed that 70 % of the tumor volume consisted of dysgerminoma, while the remaining 30 % was composed of yolk sac tumor components. The tumor was confined to the ovary, with no evidence of capsular breach, and all peritoneal and omental biopsies were tumor-free (Fig. 4).

The patient's postoperative recovery was uneventful. Serum markers were reassessed, revealing persistently elevated AFP levels at 714.20 IU/mL. One month after surgery, she underwent ovarian stimulation using an antagonist/Merional protocol, followed by transvaginal ultrasound-guided cryopreservation of seven mature oocytes through vitrification. This approach preserved her hymenal integrity, aligning with her cultural and religious beliefs as an Arab-Muslim patient.

**Fig. 5:**
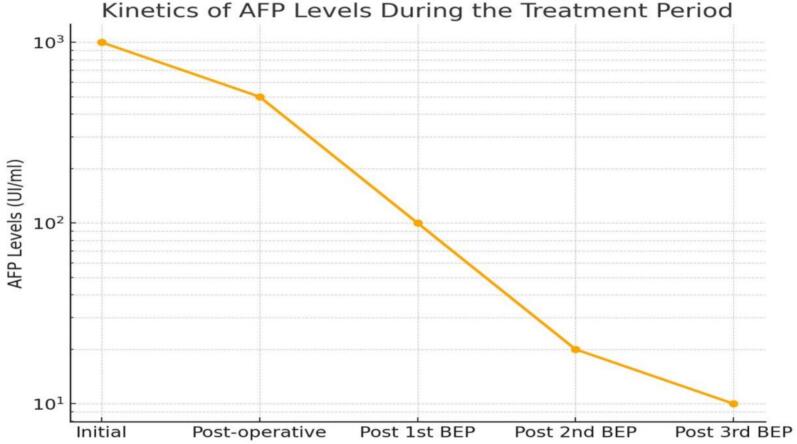
Graph representing the evolution of the patient's alpha-fetoprotein (AFP) levels throughout the treatment.

Adjuvant therapy consisted of three cycles of BEP (Bleomycin, Etoposide, Cisplatin) polychemotherapy, administered at 21-day intervals, with each cycle comprising five sessions. The patient remains under clinical, biological, and radiological follow-up (Fig. 5: AFP kinetics).

A major limitation of this case report is the rarity of mixed germ cell tumors, which limits the generalizability of the findings and conclusions to similar clinical presentations.

## 3 Patient perspectives

The patient emphasized her desire to preserve fertility while adhering to her personal beliefs. She expressed satisfaction with the option of oocyte cryopreservation, as it provided her with the possibility of future motherhood.

## 4 Discussion

Ovarian mixed germ cell tumors (OMGTs) are malignant neoplasms composed of two or more germ cell types [4]. These rare tumors account for only 8 % of germ cell tumor cases and are notably aggressive [6]. OMGTs can occur at any age, with peak incidence in adolescence. They typically present as a unilateral ovarian mass, although bilateral involvement has been reported, with incidence rates ranging between 3 % and 19 % [4].

According to G. Parikshaa et al., chronic pelvic pain and abdominal distension are the presenting symptoms in 53.3 % of cases [7]. The presence of an abdominal mass may indicate the disease and can lead to functional impairments due to compression of the urinary tract, resulting in lumbar pain, renal colic, or urinary infections, or the digestive tract, leading to constipation or partial bowel obstruction. In some instances, acute respiratory insufficiency may also occur. Approximately 10 % of cases present acutely, resembling a surgical emergency, with complications such as intraperitoneal rupture or acute ischemia due to ovarian torsion [[1], [2], [3], [4],^6^].

Abdominopelvic ultrasound is the first-line imaging modality, as it is accessible, cost-effective, non-irradiating, and effective for evaluating the nature of adnexal masses. MRI provides superior detail of the ovaries, typically identifying a large, hypervascular, multilocular solid mass with fibrous, vascularized septations [8]. CT imaging further delineates tissue characteristics and tumor relationships with adjacent organs, aiding in staging.

Any malignant ovarian tumor in a young female should raise suspicion for a germ cell tumor, prompting an assessment of specific tumor biomarkers. The Royal College of Obstetricians and Gynecologists (RCOG) recommends measuring AFP, hCG, and LDH levels in women under 40 presenting with features suggestive of OGCT [9]. The American National Academy of Clinical Biochemistry advises using only AFP and hCG, as these markers have been thoroughly studied and validated. In OMGTs, biomarker levels often correlate with disease stage and survival outcomes, depending on the tumor's cell types and proportions.

The association between elevated AFP levels in yolk sac tumors at diagnosis and prognostic significance remains debated. Some studies have shown that AFP levels >1000 IU/L correlate with a higher risk of relapse, whereas others have found no significant association between preoperative AFP levels and prognosis. Postoperative reductions in AFP levels effectively indicate residual disease, and normalization of AFP levels is crucial for evaluating chemotherapy response [1].

The treatment strategy for malignant OMGTs is relatively standardized. After staging with cytology and peritoneal biopsies, conservative treatment with unilateral adnexectomy is preferred for patients desiring future fertility. For patients not seeking fertility preservation, radical treatment with total hysterectomy and bilateral adnexectomy is standard. Pelvic and para-aortic lymphadenectomy is recommended only if lymphadenopathy is observed intraoperatively or on imaging.

Without adjuvant chemotherapy, OMGTs have a high recurrence risk. Platinum-based adjuvant chemotherapy is well established in significantly improving survival, achieving remission rates of up to 90 % [10]. The BEP (Bleomycin, Etoposide, Cisplatin) protocol is considered the gold standard due to its efficacy, low toxicity, and fertility-preserving potential [[1], [2], [3], [4],[6], [7], [8], [9]]. This protocol generally consists of three 21-day cycles following complete initial surgery, or four cycles if residual postoperative tumor remains [11]. Tumor size >10 cm and the presence of yolk sac components are associated with a poorer prognosis.

Clinical, biological, and radiological follow-up is required every three months during the first two years, every six months from years three to five, and annually thereafter. Monitoring AFP levels is a highly sensitive indicator for tumor recurrence [8]. Elevation in AFP levels, even in the absence of clinical signs, should be considered an early indication of relapse, with AFP monitoring demonstrating greater sensitivity than CT in recurrence detection [4].

## 5 Conclusion

Malignant OMGTs are exceptionally rare, and this diagnosis should be considered in any young female presenting with a rapidly enlarging abdominopelvic mass. Ultrasound remains the first-line imaging modality, while MRI provides superior mass characterization, and CT aids in staging. Serum biomarker testing is essential for confirming a germ cell origin and for post-therapy monitoring. The therapeutic approach prioritizes surgical management, followed by adjuvant chemotherapy with the BEP protocol, with an emphasis on fertility preservation whenever possible.

## Consent

Written informed consent was obtained from patients to publish this report in accordance with the journal's patient consent policy.

## Ethical approval

I declare on my honor that the ethical approval has been exempted by my establishment.

## Guarantor

Not applicable.

## Research registration number

Not applicable.

## Provenance and peer review

Not commissioned, externally peer reviewed.

## Source of finding

None.

## Funding

The authors received no funding for this work.

## Declaration of competing interest

The authors declare that they have no conflicts of interest.
